# The Pivotal Role of DNA Repair in Infection Mediated-Inflammation and Cancer

**DOI:** 10.3389/fmicb.2018.00663

**Published:** 2018-04-11

**Authors:** Ayse Z. Sahan, Tapas K. Hazra, Soumita Das

**Affiliations:** ^1^Department of Pathology, University of California, San Diego, San Diego, CA, United States; ^2^Department of Internal Medicine, University of Texas Medical Branch, Galveston, TX, United States

**Keywords:** bacterial infection, commensal bacteria, DNA damage, inflammation and cancer, *Fusobacterium nucleatum*, *Helicobacter pylori*

## Abstract

Pathogenic and commensal microbes induce various levels of inflammation and metabolic disease in the host. Inflammation caused by infection leads to increased production of reactive oxygen species (ROS) and subsequent oxidative DNA damage. These in turn cause further inflammation and exacerbation of DNA damage, and pose a risk for cancer development. *Helicobacter pylori*-mediated inflammation has been implicated in gastric cancer in many previously established studies, and *Fusobacterium nucleatum* presence has been observed with greater intensity in colorectal cancer patients. Despite ambiguity in the exact mechanism, infection-mediated inflammation may have a link to cancer development through an accumulation of potentially mutagenic DNA damage in surrounding cells. The multiple DNA repair pathways such as base excision, nucleotide excision, and mismatch repair that are employed by cells are vital in the abatement of accumulated mutations that can lead to carcinogenesis. For this reason, understanding the role of DNA repair as an important cellular mechanism in combatting the development of cancer will be essential to characterizing the effect of infection on DNA repair proteins and to identifying early cancer biomarkers that may be targeted for cancer therapies and treatments.

## Introduction

The significance of cancer as a disease that affects a large percentage of the world population is undeniable. It is one of the leading causes of death worldwide and according to World Health Organization, it causes 8–9 million deaths/year. In the United States alone, it is projected that 39.6% of the population will have some type of cancer during their life. Consequently, there are enormous expenditures in the field of cancer care. The expenditures for cancer care in the United States were nearly $125 billion in 2010 and could reach $156 billion in 2020; as mentioned in the website of National Cancer Institute. Infection and infection-associated inflammation is the major threat of cancer. Chronic inflammation from infection causes abnormal immune response, obesity, DNA damage and cancer. The best example is the inflammatory bowel disease where Ulcerative colitis and Crohn's disease develop colon cancer.

In 1863, Rudolf Virchow was the first scientist to link inflammation with cancer. He mentioned that the origin of cancer was at sites of chronic inflammation and a group of irritants with tissue injury causes cell proliferation (Balkwill and Mantovani, 2001). How does inflammation initiate malignancies? The possible answer to this question is the infection associated with chronic inflammation. Approximately 20% of cancer worldwide is caused by infection (Kuper et al., 2000; Parkin, 2006). The last 20 years of research shows that microbial infection is associated with cancer and can induce cancer progression. According to the estimate of the International Agency for Research on Cancer (IARC) approximately 18% of cancer are associated to infectious diseases that are caused by bacteria, viruses, and parasites. The well-known examples are human papilloma viruses (HPV; causing anogenital cancers), *Helicobacter pylori* (gastric cancers), hepatitis B and C viruses (hepatic cancers), and *Fusobacterium* (Colon cancer). The detailed list of microbes that have been researched in relation to their effect on certain DNA repair proteins are added in the Table 1. The gaps in knowledge in this field are reflected in the table, where there are many pathogen-associated cancers that have not been studied in relation to DNA repair proteins.

**Table 1:** A compilation of bacteria, virus, and parasite-associated cancers with some of the available information on their link to BER, NER, and MMR protein expression or mutations.

Bacteria-associated cancers	Gastric cancer	*Helicobacter pylori*		
		BER	NER	MMR
		APE1 (Ding et al., 2004)		MSH2 and MLH1 (Kim et al., 2002)
				MLH1, MSH2, MSH3, and MSH6 (Santos et al., 2017)
	Colon cancer	Enteropathogenic *Escherichia coli*		
		BER	NER	MMR
				MSH2 and MLH1 (Maddocks et al., 2013)
		*Streptococcus bovis* (Ellmerich et al., 2000)		
		BER	NER	MMR
		*Fusobacterium nucleatum* (Repass et al., 2016)		
		BER	NER	MMR
	Lung cancer	*Mycobacterium tuberculosis* (Wu et al., 2011)		
		BER	NER	MMR
		*Chlamydia pneumoniae* (Koyi et al., 2001)		
		BER	NER	MMR
	Bladder cancer	*Schistosoma hematobium* (Jemal et al., 2011)		
		BER	NER	MMR
		8-oxo-dG (Salim et al., 2008)		
		APE1 (Salim et al., 2008)		
		*Salmonella enterica* serovar Typhi (Dutta et al., 2000)		
	Ovarian cancer	*Chlamydia trachomatis* (Xie et al., 2017)		
		BER	NER	MMR
		*Mycoplasma genitalium*		
		BER	NER	MMR
Virus-associated cancers	Cervical cancer	Human Papilloma Virus (HPV) (Bosch et al., 1995)		
		BER	NER	MMR
		XRCC1 (Bajpai et al., 2016)	ERCC2 (Bajpai et al., 2016)	hMLH1 (Ciavattini et al., 2005)
		APE-1 (Shekari et al., 2008)	ERCC4 (Bajpai et al., 2016)	hMSH2 (Ciavattini et al., 2005)
			ERCC5(Joo et al., 2016)	hMLH3 (Ye et al., 2014)
	Head and neck (Oropharyngeal) Squamous cell cancer	Human Papilloma Virus (HPV) (Hajek et al., 2017)		
		BER	NER	MMR
		XRCC1 (Nickson et al., 2017)	ATR (Maddocks et al., 2013)	
		DNA polymerase β (Nickson et al., 2017)	ERCC1 (Langer, 2012)	
		PNKP (Nickson et al., 2017)		
		PARP-1 (Nickson et al., 2017)		
	Hepatocellular Carcinoma	Hepatitis B Virus (HBV) (El-Serag, 2012; Lu et al., 2017)		
		BER	NER	MMR
		XRCC1 (Almeida Pereira Leite et al., 2013; Bose et al., 2013)	TFIIH (Arbuthnot and Kew, 2001; Qadri et al., 2011)	
		OGG1 (Bose et al., 2013)	TFIIB (Arbuthnot and Kew, 2001)	
		TDG (van de Klundert et al., 2012)	XPB (Jia et al., 1999; Arbuthnot and Kew, 2001)	
			XPD (Jia et al., 1999; Arbuthnot and Kew, 2001)	
			XAP-1 (DDB1) (Sohn et al., 2000)	
		Hepatitis C Virus (HCV) (El-Serag, 2012; Lu et al., 2017)		
		BER	NER	MMR
		Neil1 (Higgs et al., 2014)	XPD (Gulnaz et al., 2013)	hMSH2 (Helal et al., 2010)
		XRCC1 (Gulnaz et al., 2013)		hMLH1 (Helal et al., 2010)
		XRCC3 (Gulnaz et al., 2013)		
	Nasopharyngeal Cancer	Epstein-Barr Virus (EBV) (Huang et al., 2017)		
		BER	NER	MMR
			XPA (Fu et al., 2015)	
	T Cell leukemia	Human T Lymphotropic Virus Type I (HTLV-1) (Liao, 2006)		
		BER	NER	MMR
		XRCC5 (Ng et al., 2001)	ERCC5 (Ng et al., 2001)	
	Kaposi sarcoma	Kaposi sarcoma-associated Herpes Virus (KSHV or HHV8) (Liao, 2006)		
		BER	NER	MMR
		APE1 (Zhong et al., 2017)		
		UNG2 (Verma et al., 2006)		
Parasite-associated cancer	Urinary bladder cancer	*Schistosoma haematobium* (Kawanishi et al., 2016)		
		BER	NER	MMR
	Cholangio carcinoma	*Opisthorchis Viverrini* (Kawanishi et al., 2016)		
		BER	NER	MMR
				hMSH2 (Liengswangwong et al., 2006)
				hMLH1 (Liengswangwong et al., 2006)

*There are many gaps in knowledge in this field, particularly in the field of bacteria-associated cancers and regulation of various DNA repair pathway proteins*.

As cited in the press release of the Nobel Assembly (Marshall and Warren, 1984, 2005): “Many diseases in humans such as Crohn's disease, ulcerative colitis, rheumatoid arthritis, and atherosclerosis are due to chronic inflammation. The discovery that one of the most common diseases of mankind, peptic ulcer disease, has a microbial cause has stimulated the search for microbes as possible causes of other chronic inflammatory conditions. Even though no definite answers are at hand, recent data clearly suggest that a dysfunction in the recognition of microbial products by the human immune system can result in disease development. The discovery of *Helicobacter pylori* has led to an increased understanding of the connection between chronic infection, inflammation, and cancer.”

**Figure 1:**
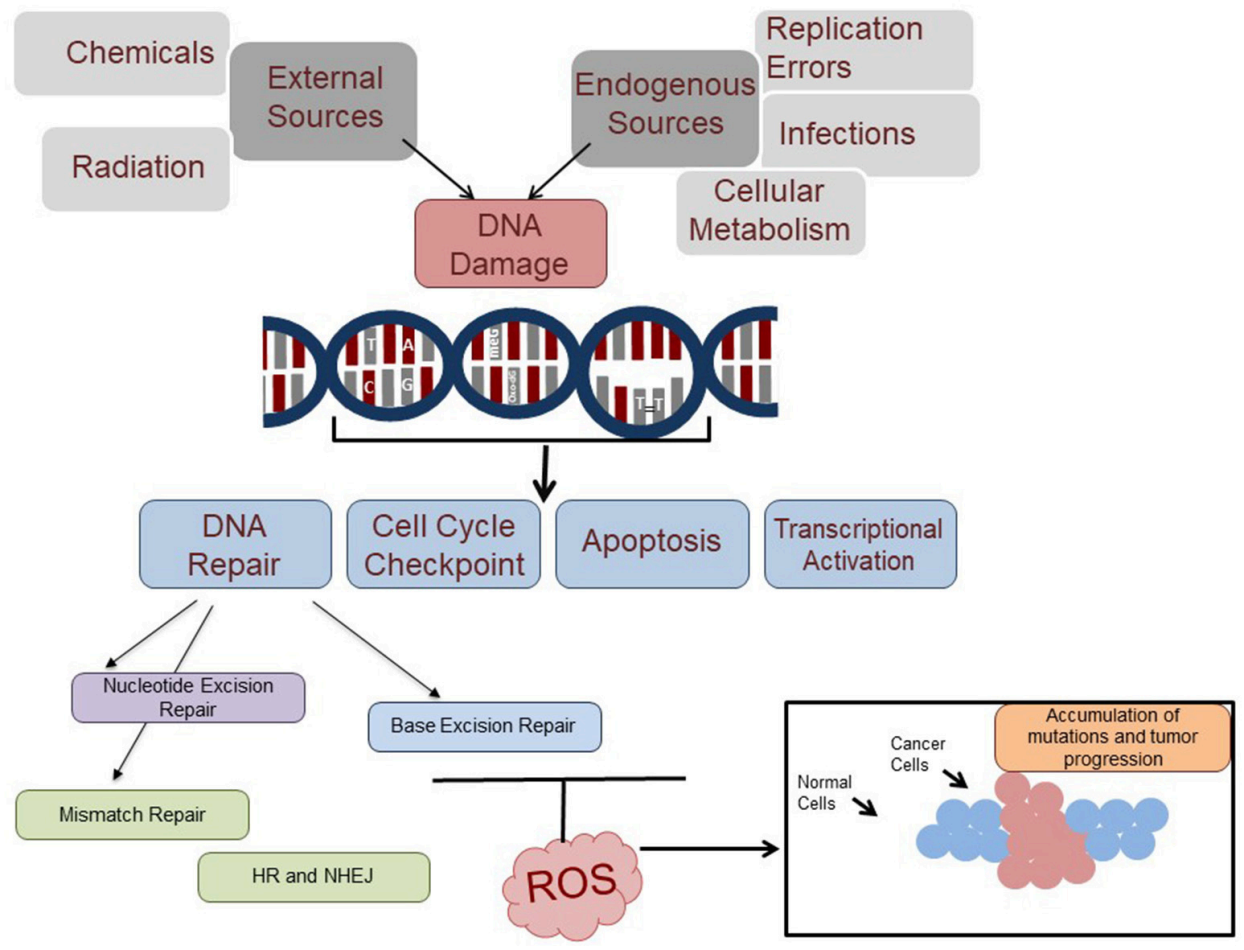
DNA damage response and the potential role of ROS in inhibition of DNA repair. DNA damage is induced by external (various environmental pollutants, chemicals, and radiation), and internal resources (infection, cellular metabolism and replication errors). These DNA damages affect the cell cycle check point, apoptosis, transcriptional activation and cancer. Part of the DNA damages (DNA base adducts, mismatch bases, damaged bases, and double strand breaks) are repaired by the nucleotide excision repair, mismatch repair, base excision repair, and homologous recombination (HR) and non-homologous end joining (NHEJ) pathways, respectively. Many of the DNA damages are ROS-induced and recognized by the BER pathway which excises and repairs the lesions. However, ROS may potentially inhibit repair through down-regulation of certain initial proteins in the BER pathway, which can cause a buildup of carcinogenic mutations and ultimately lead to tumor progression.

Chronic infection generates a milieu of inflammatory cytokines that leads to inflammatory microenvironment, a critical modulator of carcinogenesis. The persistent infection and chronic inflammation changes somatic cells by the influence of associated microbes and epigenetic factors (Fernandes et al., 2015). Hanahan et al. showed that genome instability and inflammation are the emerging hallmarks associated with cancer (Hanahan and Weinberg, 2011). Figure 1 shows the responsive elements that can trigger carcinogenesis. Bacterial infections increase cancer risk through either an extrinsic pathway, linked to induction of chronic inflammatory diseases that can increase cancer risk, or an intrinsic pathway, which is the accrual of genetic mutations that cause inflammation and transformation (Mantovani et al., 2008). Chronic inflammation has been associated with multiple types of cancer to the extent that inflammation period has been linked to increased risk of carcinogenesis (Shacter and Weitzman, 2002). Chronic inflammation is able to adjust the tumor microenvironment with cells such as tumor associated macrophages and various inflammatory agents such as chemokines, to regulate both tumor growth and angiogenesis (Coussens and Werb, 2002). Inflammation is also able to induce growth factors that serve several roles in carcinogenesis and tumorigenesis (Hanahan and Weinberg, 2011). The intrinsic pathway of genome alterations caused by infection is often linked to inflammation-mediated reactive oxygen species (ROS) production, which can increase the rate of genetic mutations that can accumulate to cause cancer (Hanahan and Weinberg, 2011).

Bacterial infection causes inflammatory response and the ROS generated by bacterial infection often results in genomic instability (Chumduri et al., 2016). This implicates a bacterial infection in compromising or at least impeding some of the several cellular mechanisms for maintaining genetic integrity and repairing mutations. As genomic instability is an underlying factor in almost all cancer cells, the link between infection and cancer development and progression is a significant one. This review will focus on the mechanism of inflammation and ROS production post-infection, and then elaborate on the genomic instability induced by infection/inflammation by discussing the effect on various DNA repair pathways. As a major focus, we will bring the link of *Helicobacter pylori* with gastric cancer and microbial infection associated colorectal cancer under the scope of DNA damage repair following infection.

## Microbial infection-mediated inflammation linked to cancer

Innate and adaptive immune responses are important to protect self from pathogenic microbial attack. Understanding of the infection process is important as bacterial and viral infection induces the inflammation that increases cancer risk (de Martel and Franceschi, 2009). The innate and adaptive immune responses will be discussed in the next section.

### Innate immune response

The immune response following recognition and invasion of microbes such as bacteria and viruses split into the innate and adaptive responses. Recognition and the initial precautionary actions are taken by the pattern recognition receptors (PRRs) of innate immune system (Pasare and Medzhitov, 2004; Akira et al., 2006). PRRs are found on the surfaces of epithelial cells and several immune cells to recognize structurally conserved pathogen-associated microbial products (PAMPs). For example, Toll-like receptor 4 (TLR4) is a PRR that binds lipopolysaccharide (LPS) on the outer membrane of gram-negative bacteria; TLR2 binds bacterial lipoproteins and lipoteichoic acids. The cytosolic sensor; NOD-like receptors (NLRs) detect intracellular pathogens. The innate immune response is the immediate mechanism by which the host attempts to clear an infection. Most of the PRRs lead to activation of MYD88-dependent pathways that eventually lead to NF-κB activation. This leads to further production of inflammatory cytokines and chemokines as well as antimicrobial peptides. Like TLRs, NLRs lead to an inflammatory response from both this signaling cascade and through the activation of caspases that act on cytokines that mediate the rest of the inflammatory response (Zarember and Godowski, 2002; Barton and Medzhitov, 2003; Basset et al., 2003; Tsung et al., 2007; Church et al., 2008; Martinon et al., 2009; West et al., 2011b). The PRRs mediated inflammatory response is initiated by various inflammatory cytokines and chemokines, which draw macrophages and mast cells that release inflammatory mediators to recruit neutrophils and plasma proteins. Neutrophils phagocytose the pathogen and surrounding debris. Phagolysosomes form through the fusion of phagosomes with granules consisting of enzymes and ROS that can kill the phagocytosed pathogen. The neutrophils can release these toxic granules and cause collateral damage to the surrounding tissue (Segal, 2005; Medzhitov, 2008; Serhan et al., 2008).

**Figure 2:**
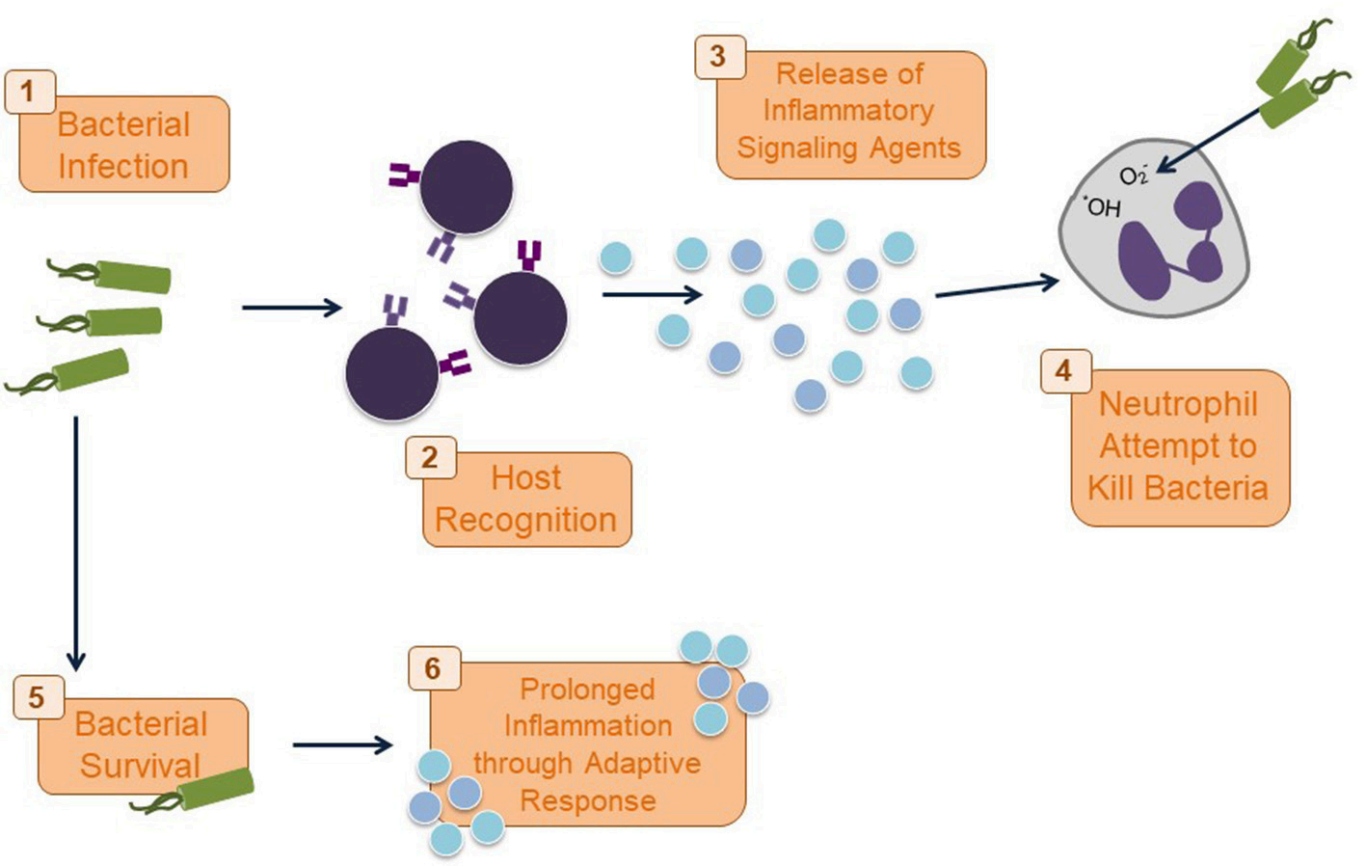
Immune response to bacterial infection. Once a bacterial infection is recognized by PRRs on host cells as part of the innate immune response, inflammatory cytokines and chemokines are released to draw neutrophils to the site of infection. Neutrophils phagocytose and kill the bacteria with ROS. If this initial response does not kill the bacteria, the adaptive immune response kicks in and may result in a chronic inflammatory response.

The increase in the pro-inflammatory cytokines will clear the bacteria and the release of pro-inflammatory agents will be halted (Figure 2), when the tissue repair phase will be started. The tissue damage and debris resulting from the neutrophil activity may prevent impedance of inflammatory response and lead to continued chronic inflammation (Nathan, 2002). Various non-degradable components of the eliminated pathogen also contribute to a lasting inflammatory response even after the threat of the invading pathogen has been erased (Medzhitov, 2008).

### Adaptive immune response

If the innate immune response is not adequate to kill the pathogen, the adaptive immune response is activated by continued inflammation at the site of infection (Zarember and Godowski, 2002; Martinon et al., 2009). The microbes must then be cleared by specialized lymphocyte (B and T-cell)-mediated mechanisms (Medzhitov, 2008). For this reason, the adaptive immune response requires time to be initiated. Prolonged infection can lead to continuous tissue damage and a chronic inflammatory response that results in various diseases. For instance, in the intestinal tract, inflammatory bowel diseases such as Crohn's disease and ulcerative colitis may manifest due to conditions of chronic inflammation that result from excessive immune activation exacerbating the initial inflammatory response to a foreign or commensal microbe (Cong et al., 2002; Macdonald and Monteleone, 2005). Specifically in Crohn's disease, the adaptive immune response plays a role through an extreme CD4 T helper type I response to overexpression of various inflammatory cytokines (Macdonald and Monteleone, 2005).

## Mechanisms that link inflammation to cancer progression

The cytokines and chemokines from innate and adaptive immune cells direct the progression of the tumor microenvironment. Downstream effectors NF-κB, AP-1, STAT control the inflammatory milieu either by affecting tumor survival, growth, and tumor progression by signaling for molecules such as IL-6, IL-23 or by anti-tumor immunity (IFN-γ, IL-12). The inflammatory cytokines have been reported to generate ROS and reactive nitrogen intermediates (RNI) using NADPH oxidase in phagocytic cells and epithelial cells (Yang et al., 2007). These ROS are the major source of damage to nucleic acid, proteins and lipids.

### Reactive oxygen species (ROS)

ROS refer to various, highly reactive and partially reduced metabolites of oxygen such as H_2_O_2_ that are essential signaling molecules in the human immune system (Martindale and Holbrook, 2002; West et al., 2011b). ROS includes oxygen radicals (superoxide, hydroxyl, peroxyl and alkoxyl) and certain nonradicals that are either oxidizing agents and/or are easily converted into radicals, such as HOCl, ozone, peroxynitrite, singlet oxygen and hydrogen peroxide. ROS initiates DNA base oxidation which, if not repaired properly, may lead to induce a mutation.

Inflammatory cytokine signaling also depend on ROS. ROS is also crucial for inflammasome signaling and increased mitochondrial ROS activate the NLRP3 inflammasome (Zhou et al., 2011). Additionally, the post-translational modifications that ROS are associated with are often linked to modifications in protein cysteine residues that are generally associated with either Ca^2+^ mediated signaling or tyrosine phosphorylation. This implicates ROS in cell motility, mitosis, differentiation, and immune response or regulation (van der Vliet, 2008). The immediate effect of excessive ROS presence in the host is a chronic inflammatory state that exacerbates both inflammation and, as a result, ROS production. Also, cancer cells utilize mROS to constitutively activate proliferation pathways to promote tumor growth (Cairns et al., 2011). Inability of cellular antioxidants to curtail ROS leads to oxidative stress on host cells that can lead to many adverse effects including induction of DNA damage (Ernst, 1999). Therefore, following oxidative stress, the cell must survive by either adapting to the induced stress or by repairing the damage (Martindale and Holbrook, 2002). Inability to repair or adjust to the damage will lead to chronic conditions such as cancer, diabetes, and various neurological or cardiovascular diseases. The specific mechanisms of DNA damage repair that diminish the oxidative damage done by ROS will be discussed later in this review.

ROS in immune cells are important to kill extracellular pathogens by using the NADPH oxidase in phagosomes. Despite the various positive roles of ROS in immunity and other processes, they are associated with conditions such as diabetes, hypertension, cancer, and autoimmune diseases due to their ability to change and damage cellular proteins, lipids, and DNA (Zimmerman and Cerutti, 1984; Guzik et al., 2007). ROS is acquired through both exogenous and endogenous sources. Exogenous sources of ROS include carcinogen induced or generated ROS; for example, xenobiotics, chlorinated compounds, and radiation are associated to oxidative stress. The main endogenous source of ROS in the human body is through mitochondrial respiration or Ox-Phos (Oxidative phosphorylation) system. The electron transport chain uses mitochondrial oxidative phosphorylation complexes that lead to generation of ROS that can potentially harm cellular components such as proteins and nucleic acids through post-translational modifications and oxidation (Molina-Cruz et al., 2008). ROS produced by Ox-Phos pathway participate in immune signaling with TLR and cytosolic RIG-I like receptors (RLRs). West et al. reported that stimulation of cell-surface TLRs (TLR1, TLR2, and TLR4), but not endosomal TLRs (TLR3, TLR7, TLR8, and TLR9), leads to an increase in mROS (Mitochondrial Reactive Oxygen Species) production through TRAF6 and ECSIT signaling (West et al., 2011a). Mitochondrial ROS enhance RLR signaling in autophagy-dependent Atg5-depleted cells that indicate the importance of autophagy in innate antiviral defense (Tal et al., 2009).

The most significant damages caused to DNA due to high concentrations of ROS include double and single strand breaks, oxidized DNA bases, and aberrant DNA cross-linking. Each type of ROS plays a different role in inflicting damage. For example, hydrogen peroxide (H_2_O_2_) directly induces DNA damage. The versatility of different ROS is reflected in the wide array of DNA damage that they can cause. In general, the hydroxyl molecule (OH^−^) is the most damaging form of ROS, but other forms like the oxygen molecule (O_2_), RO_2_, and RO are also capable of different types of damage. For instance, while OH^−^ can react with all of the bases and the deoxyribose backbone of DNA, O_2_ preferentially targets guanine residues (Wiseman and Halliwell, 1996; Valko et al., 2007; Imlay, 2008). In a wider scope, ROS are generally likely to react with and damage DNA through oxidation, methylation, depurination, and deamination. The lesion most often found due to oxidative DNA damage, 8-hydroxyguanine (8-OHdG) or the nucleoside 8-hydroxydeoxyguanosine, is generally considered as marker of oxidative damage incurred by a cell. For instance, many studies examined for increased production of ROS culminating in an increased level of oxidative DNA damage by measuring 8-hydroxyguanine (Dandona et al., 1996; Farinati et al., 1998). In another study, it was observed that P53 acts as a defense against ROS-mediated DNA oxidation in various experimental conditions by detecting the presence of 8-oxodeoxyguanosine in the DNA (Sablina et al., 2005). The 8-hydroxydeoxyguanosine and other oxidized DNA lesions (8-oxo-adenine, thymine glycol, 5-hydroxy-deoxycytidine) have also been observed in many mutated oncogenes and tumor suppressor genes and these lesions are able to induce further neoplastic mutations in the DNA. The presence of high levels of 8-oxoguanine lesions, along with many other oxidative lesions, was shown in the DNA of tumorous tissues from many patients with different types of cancers (Klaunig and Kamendulis, 2004). This indicates a serious implication of oxidative stress and oxidative DNA damage in carcinogenesis (Figure 1).

## Involvement of microbes in ROS-linked carcinogenesis

The gut microbiota supplement a significant portion of the human metabolism, and the composition and activity of the microbiota plays a large role in susceptibility to metabolic diseases such as hyperglycemia, hyperlipidemia, insulin resistance, and obesity (Vijay-Kumar et al., 2010; Spencer et al., 2011). Microbes protect themselves from the ROS generated by host using an enzyme called superoxide dismutase (Sod), which is abundant in cells throughout the body. This enzyme attaches (binds) to molecules of copper and zinc to break down toxic, charged oxygen molecules called superoxide radicals. Interestingly, bacteria has protective proteins such as SodA, SodB, SodC, AhpCF, KatG, KatE to detoxify ROS and proteins to counter damage (e.g., SoxRS, OxyRS, and SOS regulons) (Imlay, 2008). On the host side, Nox2, present in the NADPH complex is responsible for the generation of ROS (Lambeth, 2004). The generation of ROS is important for host defense as patients with chronic granulomatous disease (CGD) with deficiencies in NOX2 components are susceptible to infection (Cross et al., 2000).

Transcription factors vital to cellular processes such as inflammation, cell cycle regulation, motility, and growth like NF-κB, p53, HIF-1α, β-catenin/Wnt can be activated by the oxidative stress caused by an imbalance in the presence of oxidative ROS and the countering antioxidants. By mediating activity of genes and proteins related to oxidative stress, ROS are able to affect further cellular properties, such as cell growth, differentiation, and apoptosis, which can induce transformation (Reuter et al., 2010). For instance, ROS produced as a result of exposure of mouse mammary epithelial cells to MMP-3, a stromal enzyme that is linked to inducing epithelial-mesenchymal (EMT) transition and malignant transformation, caused activation of the transcription factor SNAIL, which induced oxidative DNA damage and EMT (Radisky et al., 2005). ROS-induced oxidative stress can also affect gene expression through direct DNA (de)methylation, which is an epigenetic method of silencing and activating certain genes by changing the physical accessibility of certain genes (Klaunig and Kamendulis, 2004). Chromosomal alterations induced by ROS can also lead to cellular damage. The genomic instability and transcriptional changes that accompany ROS and oxidative stress can therefore lead to carcinogenesis.

Post-infection inflammation-mediated mechanisms that assist in tumor formation and progression are an indirect method of bacterial infection leading to cancer. There are also several factors in a bacterial infection that can directly induce DNA damage or alter cell-signaling pathways that can lead to carcinogenesis. For instance, several bacteria such as *Escherichia coli* and *Shigella dysenteriae* are able to produce genotoxins (colibactin and shiga toxin, respectively) that inflict damage on the host DNA such as DNA strand breaks that may affect tumor suppressors or oncogenes (Gagnaire et al., 2017).

## DNA repair mechanisms

**Figure 3:**
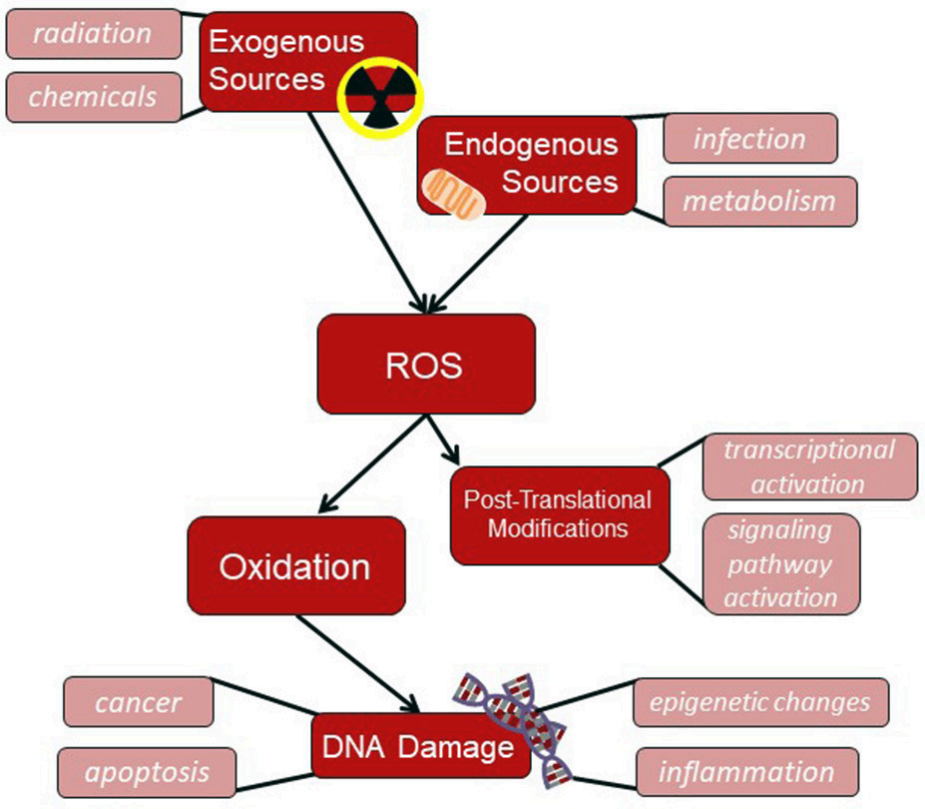
ROS flowchart. ROS produced by either exogenous sources, such as radiation, or endogenous sources, such as through cellular mitochondria, can induce DNA damage through oxidation or cause post-translational modifications on cellular proteins.

Cellular DNA is altered either during replication or by external mutagens. Misincorporation of DNA bases can occur during replication; however, it is combatted through the proofreading activity of DNA polymerases. There may however be errors during DNA replication that are not recognized by the polymerases. There is also a great wealth of mutagens that can cause extensive changes in the sequence of human DNA. Of these, just oxidative DNA damage is estimated to arise about 10^5^ times in 1 day due to ROS-induced damages (Lengauer et al., 1998). Accumulation of all these mutations would severely inhibit the ability of cells to survive and/or maintain proper cellular functions in almost all cases. Fortunately, the cell has several mechanisms that are specialized to recognize and repair different types of DNA damage. Of these, this review will describe the mechanisms that address alterations in DNA sequence and their link to various cancers, including mismatch repair (MMR), nucleotide excision repair (NER), base excision repair (BER), and homologous recombination (HR) and non-homologous end joining (NHEJ). Most importantly, MMR, BER, HR, and NHEJ alterations have been linked to chronic inflammatory states (Figure 3). Therefore, the focus will be on genetic instabilities induced through deregulation of these repair pathways, in some cases due to inflammation, that lead to genetic instabilities that may contribute to cancer formation.

DNA repair pathways recognize and correct mismatches present in the DNA, abnormal bases, single-stranded and double-stranded DNA breaks (DSBs). The MMR, BER, and NER pathways respond to specific lesions in DNA residues. DSBs are particularly dangerous lesion and are repaired by two principal pathways: NHEJ pathway functions in all phases of the cell cycle, while the high-fidelity HR pathway requires a template for repair and utilizes available sister chromatids during the S and G2 phases of the cell cycle.

### Nucleotide excision repair

Nucleotide excision repair, or NER, is a versatile repair pathway that recognizes bulky adducts and general base lesions that cause a distortion of the double-helix structure of DNA. The major sources of these types of DNA damage are ultraviolet radiation and various types of genotoxic chemicals (Hoeijmakers, 2001). These lead to lesions such as pyrimidine dimers, cyclobutane pyrimidine dimers (CPD), and 6–4 photoproducts. NER-mediated pathway employs several different proteins to carry out a multi-step “cut-and-patch”-like pathway (Shuck et al., 2008). The defect in NER generates human genetic disorders and the bulky adducts targeted by NER mechanism can block replication and/or transcription which can lead to apoptosis or necrosis (Sancar et al., 2004).

The NER mechanism is divided into two pathways that have different methods of lesion recognition. These are the global-genome (GG-NER) and transcription-coupled repair (TC-NER) pathways. In GG-NER, DNA damage is removed from the whole genome while TC-NER is primarily involved in repairing the damage on the coding strand of actively transcribed genes (Hoeijmakers, 2001; Jackson and Bartek, 2009). Both pathways differ in the initial recognition steps. In GG-NER, the major proteins involved in the recognition are the XPC/HR23B/CEN2 (XP complementation group C/Rad23 homolog B/Centrin-2) protein complex. TC-NER is important to protect the cells from UV-light-induced apoptosis. In TC-NER, the damage is recognized and RNA Pol II stalls, Cockayne syndrome protein CSB transiently interact with RNA Pol II and the other associated proteins can take care of the damage to repair. In patients with Cockayne syndrome have defective TC-NER (Sancar et al., 2004; Fousteri and Mullenders, 2008; Hanawalt and Spivak, 2008).

### Mismatch repair

Mismatch repair, or MMR, fixes mismatched base pairs and insertion-deletion loops that are generally a product of incorrect genomic DNA replication. MMRs have the ability to inflict serious damage on the cell without killing it since they may go unnoticed and accumulate (Kunkel and Erie, 2005). MMR deficient cells can display a mutator phenotype, characterized by microsatellite instability and an elevated mutation frequency. The germline mutations in MMR genes can lead to a variety of cancers, including the non-polyposis colon cancer/ Lynch syndrome (Peltomäki, 2001). MMR involves three steps: a recognition step for identifying the mispaired bases, an excision step to remove the error-containing and the synthesis step, where the gap is filled-in by DNA polymerases. Therefore, the MMR pathway is very important to prevent cancer.

Several MMR proteins can be regulated upon chronic inflammation through the activation of HIF-1α by inflammatory cytokines and ROS (Colotta et al., 2009). The inability of MMR to repair single base pair or small-scale mutations results in microsatellite instabilities such as poly CA repeats, present in various cancers (Lengauer et al., 1998).

### Base excision repair

Base-excision repair or BER recognizes a wide variety of damaged bases including those that underwent oxidation, alkylation, methylation, deamination, and hydroxylation (Hoeijmakers, 2001). It also repairs ROS induced strand breaks that consist of sugar fragments or 3′phosphate ends that are non-ligatable (Hegde et al., 2008). Due to its recognition and repair of an extensive range of ROS induced damages, BER is the major defense against accumulation of mutations caused by ROS. Additionally, the lesions targeted by BER are generally small-DNA base adducts. These types of lesions are more likely to be kept within the genome without cell apoptosis and can result in continued mutations in tumor suppressor and oncogenes that can lead to cancer (Hoeijmakers, 2001). Especially through this framework, BER earns greater importance in preventing cellular transformation and cancer.

Single strand break repair pathway (SSBR) is now considered a specialized sub-pathway of BER. They share several common proteins including APE1, Polβ, LIGIIIα, along with the nick sensor poly (ADP-ribose) polymerase 1 (PARP1) and the scaffold protein X-ray cross-complementation group 1 (XRCC1) (Caldecott, 2008). ROS generate 8-oxoguanine (8-oxoG), ring-opened purines (formamidopyrimidines or Fapys), and other oxidized DNA base lesions that are repaired via the DNA BER pathway. BER begins with the recognition of an altered base by a DNA glycosylase. There are two classes of DNA glycosylases, the first group with the enzymes OGG1 and NTH1 utilize an internal Lys residue as the active site nucleophile. The second group comprising of NEIL1, NEIL2, and NEIL3 use N-terminal Pro or Val as the active site (Hazra et al., 2002, 2007; Sancar et al., 2004). The first and second group have distinct structural features and reaction mechanism but have overlapping substrate specificities. The NEIL proteins are able to preferentially target single stranded DNA and also lesions from a DNA bubble while NTH1 and OGG1 only excise lesions from double-stranded DNA, as they use the second strand as a template for repair (Hegde et al., 2008). Therefore, the NEIL proteins can be functional for replication/transcription errors (Banerjee et al., 2011).

These oxidized base-specific mammalian DNA glycosylases are bifunctional. Monofunctional DNA glycosylases excise the altered base in a way that leaves behind an AP site that needs to be processed by an AP endonuclease (3′-OH and 5′dRP are generated by APE1). On the other hand, the mammalian bifunctional DNA glycosylases have an associated AP lyase activity generating 3′dRP (OGG1, NTH1, and NEIL3) or 3′-P (NEIL1 and 2) and 5′-P. The DNA polymerase then fills in the gap using the template DNA strand and finally, DNA ligase seals the nick by completing the repair of the DNA duplex (Hitomi et al., 2007; Hegde et al., 2008; Maynard et al., 2009). The essential component of BER is the DNA glycosylase that recognizes and removes the oxidized base. Inhibition of the BER proteins may lead to an accumulation of oxidized DNA damage induced by ROS and the possible escalation of mutation rate upon an inflammatory response to bacterial infection that can contribute to carcinogenesis (Maynard et al., 2009).

The fact that defects in various components of NER and MMR have been shown to contribute to certain cancers indicates that the mutations that arise from inhibition of these repair mechanisms are certainly an important causative factor to cancer. For this reason, although BER has not been studied as thoroughly as NER and MMR in the context of cancer, it may have a significant role in the perpetuation of bacterial infection and inflammation mediated cancers such as gastric cancer and, to some degree, colorectal cancer (Wallace et al., 2012; Leguisamo et al., 2017). Various human DNA glycosylases have already been implicated in various types of cancer. NEIL2 was shown to protect against the oxidative damage that is induced by secondhand smoke in human lung cells, and lower levels of NEIL2 are associated with development of lung tumors (Sarker et al., 2014). It has also been shown that knock out of NEIL2 increased the accumulation of spontaneous mutations, and a variant of NEIL2 was observed in lung cancer samples (Dey et al., 2012). Furthermore, functional variants of NEIL2 have been linked to greater risk of squamous cell carcinoma in the oral cavity and oropharynx, making it a possible marker for risk to and progression of squamous cell carcinoma in the oral cavity and oropharynx (Zhai et al., 2008). In a study of human non-small cell lung cancer, it was found that carriers who were positive for silencing of the DNA glycosylase OGG1 via methylation had a 2.25-fold higher risk of developing non-small cell lung cancer than carriers who did not exhibit OGG1 methylation (Qin et al., 2017). In another study, an OGG1 variant (Ser326Cys polymorphism) was found to increase lung cancer risk by 24% in an analysis of seven studies totaling over 3,000 cases and controls. Both the mRNA and protein expression of MUTYH, a human DNA glycosylase that repairs the foremost oxidative DNA damage in prostate cancer (8-hydroxyguanine), was down-regulated in about two thirds of prostate cancers compared to the non-cancerous prostate tissue data presented in two separate publicly available databases (Shinmura et al., 2017). The apparent links between DNA glycosylase inhibition or silencing and various cancers indicates that: (1) Oxidative damage is a major contributor to the accumulation of genetic mutations that can lead to carcinogenesis, and (2) BER plays a significant role in repressing the accumulation of ROS-induced mutations and therefore inhibition of certain BER proteins may contribute to carcinogenesis. The roles of various bacterial infections in the development of gastric and colon cancer will be discussed later to explain the relationship of bacterial infection to DNA damage and repair inhibition.

### Homologous recombination and non-homologous end joining

HR and NHEJ are mechanisms to repair double strand breaks in DNA (Jackson and Bartek, 2009). These repair processes are important since double strand breaks can be extremely harmful to the genome and are largely considered the most lethal type of DNA lesions since both strands of the DNA are affected (Helleday et al., 2008). Double strand breaks can be induced by X-rays, genotoxic chemicals, during replication of single strand breaks, or by ROS (Hoeijmakers, 2001). Due to the possibility of ROS inducing double strand breaks in the DNA, bacterial infection and the resulting inflammation are implicated in this type of DNA damage. HR is present during DNA replication in the S and G2 phase of the DNA cycle while NHEJ combats direct double strand breaks that can be induced by the other factors listed above, such as X-ray or ROS exposure, and is predominant in the G1 phase of the cell cycle (Hoeijmakers, 2001; Jackson and Bartek, 2009).

In HR, the MRN sensor complex; containing MRE11 (meiotic recombination 11), RAD50, and NBS1 recruits ATM (ataxia telangiectasia mutated), generates DNA breaks followed by phosphorylation of histone H2AX (generating γH2AX) that amplifies the damage signal (Blackwood et al., 2013). Classical non-homologous end-joining (C-NHEJ) is the major pathway for DNA double strand break repair. Depletion of C-NHEJ factors significantly abrogates double strand break repair in transcribed but not in non-transcribed genes (Chakraborty et al., 2016). In NHEJ, the Ku70-Ku80 initiates NHEJ, which are the sensor proteins that recruit DNA–PK (DNA-dependent protein kinase) and end-processing proteins, followed by ligation of the breaks by a complex consisting of DNA ligase IV/XRCC4; all the end-processing enzymes (Jackson and Bartek, 2009). Mutations in proteins that repair double strand breaks are linked to higher risk for different types of cancers, predominantly lymphomas (Hoeijmakers, 2001). Interestingly, HR occurs only in cycling cells while NHEJ occurs in all cells.

## Non-ROS linked inflammation-associated mechanisms for promoting carcinogenesis

Cancer related inflammation has been observed in most neoplastic tissues through observations of white blood cell and tumor-associated macrophage infiltration into the tumor microenvironment as well as pro-inflammatory cytokine and chemokine presence. These inflammatory factors have been linked to increased tissue remodeling and angiogenesis and are therefore stated to lead to cancer related inflammation (Balkwill and Mantovani, 2001; Colotta et al., 2009; Ostrand-Rosenberg and Sinha, 2009). Other than the intrinsic effect of cancer stimulating genomic instability induced by chronic inflammation, there are many components of an extrinsic inflammatory pathway that accelerate the further development of cancer. In this case, various pro-inflammatory cytokines, chemokines, and other inflammation-linked factors assist in tissue remodeling and angiogenesis.

For instance, inflammation activates NF-κB, which in turn activates various other inflammatory cytokines as well as angiogenic factors (Colotta et al., 2009). In the case of an acute inflammatory response of the innate immune system, the activation of NF- κB is not significant to the extent that it will affect angiogenesis and cancer progression. However, in the case of chronic inflammation, as is common in various bacterial infections such as *H. pylori* infection, the activation of these cytokines and factors will be consistent and significant enough to contribute to cancer development (Mantovani et al., 2008). For instance, the mediators that are downstream of NF-κB will help in neoplasia assisted by inflammation. Therefore, prolonged activation of NF-κB will promote tumor cell survival, initiation and progression of tumor tissue formation (Karin, 2006; Bollrath and Greten, 2009). In addition to NF-κB, various other inflammation-associated molecules such as IL-6 and TNF will aid in cancer progression through various mechanisms (Colotta et al., 2009). IL-6 specifically assists in tumor cell survival and growth. IL-6 is produced by myeloid-derived suppressor cells that, as suggested by the name, suppress T-cell activation. These cells are recruited to areas with chronic inflammation by pro-inflammatory mediators and are extremely influential in promoting cancer survival by allowing them to evade the immune system's attacks via T-cell activation. TNF, on the other hand, mediates inflammation and can promote the growth of a tumor by assisting in angiogenesis, epithelial to mesenchymal transition, and other mechanisms. This pro-inflammatory cytokine is associated with tumor-associated macrophages, which are also found in areas of chronic inflammation. When tumor-associated macrophages secrete TNF, the activation of Wnt/ β-catenin signaling pathway is promoted and this leads to greater tumor development (Colotta et al., 2009). The Wnt/ β-catenin pathway is a significant signaling mechanism that controls transcription for proteins involved in cell proliferation and cell fate determination (MacDonald et al., 2009). Moreover, in both T cells and ECs, an upregulation of the Wnt/β-Catenin pathway occurs upon infection, a pathway usually associated with changes in the cellular turnover rate, tissue regeneration and cellular metabolism (Karin and Clevers, 2016). The activation of microbe-sensing pathways by ECs, associated with similar gene expression changes in both ECs and IELs in immune-response-related and metabolic pathways, pointed to ECs as potential primary microbe-responding cells that could prompt neighboring IELs. Lastly, NF- κB activation leads to the production of proangiogenic factors like vascular endothelial growth factor (VEGF), which allow for the tumor to grow and spread to distal sites to result in metastasis (Ellis and Hicklin, 2008). Of the countless links between inflammation and cancer progression, only a few have been discussed here. Ultimately, however, it is clear that cancer related inflammation is a significant factor in both the initiation of cancer as well as the consecutive growth and metastasis of a tumor.

### Bacterial toxins can cause DNA damage

Bacteria not only generates DNA damage but also interacts with the host DDR pathways so damage cannot be efficiently repaired. Some of the pathogenic strains of bacteria produce toxins such as Cytolethal distending toxin (cdt) and Colibactin. Cdt is present in *Campylobacter jejuni, Haemophilus ducreyi, Actinobacillus actinomycetemcomitans, Shigella dysenteriae, Helicobacter cinaedi, Helicobacter hepaticus, Salmonella* species. Cdts recruit the MRN complex (MRE11/Rad50/NBS1) and generate DSBs that ultimately progress to gastro-intestinal cancer (Taieb et al., 2016). Another toxin, Colibactin is a polyketide nonribosomal peptide produced by several species of *Enterobacteriaceae;* for example in some of the *E. coli* strain with pks, *Klebsiella pneumoniae* and *Enterobacter aerogenes*. Colibactin is responsible for alkylation and interstrand crosslinks of DNA followed by generation of DSBs (Nougayrède et al., 2006).

There are multiple mechanisms for bacterial infections to induce cancer, and we focused gastric cancer and colon cancer here. Further studies in this arena will give more in depth insight to the mechanisms of association between specific bacterial infections and cancers, but in the next section we will discuss the well-known and characterized bacterial-infection associated cancers especially through the lens of ROS induced DNA damage and alteration of DNA repair pathways.

## Bacterial infection-associated with cancers

The cumulative effect of the various processes that take place after a bacterial infection, either through the direct action of the bacterium or indirectly through bacteria-induced pathways, is linked to carcinogenesis in various tissues. Many infectious agents have previously been linked to cancers, and are implicated in about 20% of human tumors (de Martel et al., 2012). For instance, respiratory tract and lung cancers have been linked to pulmonary infections caused by *Chlamydia pneumonia* and *Mycobacterium tuberculosis* (Chaturvedi et al., 2010). *Chlamydia trachomatis* and *Neisseria gonorrhoeae* infections have been associated with genitourinary cancers, and so on (Smith et al., 2004). Of the many bacterial species that have been implicated in cancer formation, *Helicobacter pylori* has been shown to play a significant role in contributing to the global gastric cancer burden and has therefore been studied more thoroughly than many other species linked to cancers. Despite a clear, demonstrated link between *H. pylori* infection and gastric cancer incidence, the exact mechanism of carcinogenesis has yet to be discovered and characterized in depth. *Fusobacterium nucleatum*, on the other hand, has been observed in large amounts in the intestinal tissues of colorectal cancer patients, but a link to cancer has not been concretely established as of yet (Gagnaire et al., 2017). Although there are some contributions available on the possible mechanisms with which it may be linked to colorectal cancer, they have not been properly defined either.

### Microbial infection-associated mechanisms to promote cancer

In mammalian cells, all the above-mentioned repair mechanisms can repair damage using the DNA damage responses (DDRs) and failure in these bring DNA damage/mutation and genomic instability. Microbial infection is one of the major reasons of the failure of DDRs.

Microbes such as bacteria, virus and parasites are able to activate or alter various signaling pathways that may lead to either activation of oncogenes or down-regulation of tumor suppressor genes in a contribution to cancer progression (Francescone et al., 2014; Sheflin et al., 2014). Examples of these modifications and pathways are discussed in this section. They also modulate repair pathways as mentioned in the table that can generate mutations linked to cancer.

*Helicobacter pylori* infection leads to activation of PI3K-AKT pathway, which ultimately leads to degradation of tumor suppressor p53. It also contributes to cell transformation and growth by both preventing the degradation of and activating β-catenin through various bacterial effectors such as VacA (vacuolating cytotoxin A) (Tabassam et al., 2009). Similarly, a virulence factor of *Fusobacterium nucleatum* called *Fusobacterium* adhesin A (FadA) is able to bind E-cadherin to induce greater β-catenin release and ultimately activate WNT signaling, which is oncogenic (Rubinstein et al., 2013). *Helicobacter pylori* and *Salmonella enterica* serovar Typhimurium both activate MAPK and AKT signaling pathways upon infection leading to altered mediation of cell growth, proliferation, migration, and other important processes relevant to cellular transformation (Sokolova et al., 2008; Gagnaire et al., 2017).

The bacterial toxins which cells are exposed when infected can alter the cell cycle and ultimately affect some of the processes, which are implicated in carcinogenesis: proliferation, apoptosis, and differentiation (Mager, 2006). Certain bacteria, which are classified as cyclomodulins, are able to change host cell cycle patterns with cell-cycle inhibitors, such as cytolethal distending toxins and cycle inhibiting factor, and cell cycle stimulators such as cytotoxic necrotizing factor (Nougayrède et al., 2005).

Bacteria of the microbiota are also implicated in cancer through their ability to construct biofilm. Biofilms form when bacteria aggregate and secrete a substance that allows them to stick to surfaces that generally have a mucosal lining (Johnson et al., 2016). In addition to being linked to inflammatory bowel conditions, bacterial biofilms have been seen on colorectal cancers, preferentially in proximal colon cancers, which have a higher mortality rate, than distal colon cancers (Dejea et al., 2014).

### *H. pylori* associated gastric cancer

*Helicobacter pylori* is a gram-negative and spiral shaped bacterium. It has flagella that assist in movement and is able to survive at very low pH, making it a main colonizer of the stomach. Infection with *H. pylori* is associated with greater susceptibility to further infections, diarrhea, and chronic gastritis (Tomb et al., 1997; Crew and Neugut, 2006). While eradication of *H. pylori* infection is possible, there is high probability of relapse as well as antimicrobial resistance in many strains of the bacteria. The standard triple antibiotic treatment of *H. pylori* cures up to 70% of infected patients since there is a growing resistance to clarithromycin. Additionally, a very large portion of the world, about half of the total population, is exposed to or infected by *H. pylori* (Parsonnet et al., 1994; Malfertheiner et al., 2012). As *H. pylori* is the greatest risk factor for gastric cancer development, it is very important to study mechanisms of carcinogenesis upon infection in order to develop possible therapeutic and treatment options that address the specific pathways altered by *H. pylori*. Studies have shown that eradicating *H. pylori* infection decreases gastric cancer development in patients without premalignant tumors and prevents malignant transformation in patients with premalignant tumors (Wong et al., 2004; Malfertheiner et al., 2012). This further reinforces the link between infection and gastric cancer.

#### Non-inflammatory pathways for cellular transformation upon *H. pylori* infection

*H. pylori* has multiple bacterial effectors that are able to alter cellular signaling pathways in favor of carcinogenesis. For instance, vacuolating cytotoxin A (VacA) and outer inflammatory protein A (OipA) are involved in epidermal growth factor receptor activation which leads to PI3K-AKT signaling and ultimately activates β-catenin (Suzuki et al., 2009; Wroblewski et al., 2010). This signaling cascade leads to transcriptional activation for cell growth. If the infection cannot be cleared, this becomes constitutively active and can then cause cellular transformation. This is just one example of how bacterial effectors can contribute to carcinogenesis in a non-inflammatory pathway. However, these effectors such as OipA are also able to induce proinflammatory cytokine expression along with other oncogenic proteins that have a significant effect on cellular transformation. Bacterial oncoproteins such as CagA are additional features of the bacteria that contribute to carcinogenesis (same reference as on line 573). The cag pathogenicity island, present in cag^+^ strains of *H. pylori* has genes which encode for type IV bacterial secretion system, commonly known as T4SS, that is able to export bacterial proteins such as CagA upon bacterial attachment to host cells. Once it enters a host epithelial cell, this protein can be activated via phosphorylation to mitigate apoptosis and induce greater cell proliferation to contribute to carcinogenesis (Polk and Peek, 2010). Despite their active role in altering many cancer-associated cellular pathways, bacterial oncoproteins and effectors are only able to increase gastric cancer risk. The ultimate development of gastric cancer also relies on chronic inflammatory response to *H. pylori* infection, which is accompanied by a wide variety of consequences that contribute to cellular transformation (Lamb and Chen, 2013).

#### Inflammation-associated pathways for cellular transformation upon *H. pylori* infection

The inflammatory response to *H. pylori* infection can significantly alter cellular signaling and activity to induce transformation through some of the many pathways already discussed in this review (Figueiredo et al., 2002). The focus here will be on DNA damage induced by *H. pylori* mediated inflammation as well as obstruction of DNA repair pathways by the bacterium.

*H. pylori* colonization is characterized by recurring infections even after assumed eradication of the bacteria as well as chronic gastritis, which signifies a continuous inflammatory response that is started as a host response to infection but is continued with the aid of the bacteria by effectors and oncoproteins that alter chemokine and cytokine release in the infected host cells (Miftahussurur et al., 2017). One such oncoprotein is Tipα, a membrane protein secreted by *H. pylori* that is associated with epithelial to mesenchymal transition by activating IL-6 cytokine-dependent STAT3 signaling and greatly impacts cancer cell invasiveness. Similar to IL-6, many other inflammatory cytokines play a role in extrcellular matrix degradation to promote cell motility and angiogenesis (Chen et al., 2017).

An accumulation of DNA damage caused by chronic inflammation and resulting ROS has the potential to induce cellular transformation. In a normally functioning cell, the multiple DNA repair pathways are able to curb the accumulation of DNA mutations by addressing the damage as it occurs. In a cell infected by *H. pylori*, it is shown that mismatch repair (MMR), the major pathway that repairs small-scale mutations such as single base pair mismatches, is inhibited (Kim et al., 2002; Santos et al., 2017). This potentially allows for seemingly minute mutations to accumulate in oncogenic or tumor suppressor genes and lead to cancer development. Two vital MMR proteins, MSH2 and MLH1, are directly affected by *H. pylori* infection. Additional MMR proteins MLH1, MSH3, MSH6, PMS1, and PMS2 have also been shown to be down-regulated upon *H. pylori* infection of gastric cell lines AGS and BG (Machado et al., 2013; Strickertsson et al., 2014; Santos et al., 2017). The various oxidative damages induced to DNA by *H. pylori* infection are supposed to be repaired by the BER pathway in a normally functioning cell. In a cell infected with *H. pylori*, there is decreased expression of vital BER proteins. APE-1, an AP endonuclease, and YB-1, an early-stage repair protein of BER, are down-regulated upon *H. pylori* infection (Machado et al., 2013). Down-regulation of the DNA glycosylase OGG1, which is very important for recognition and removal of abasic sites induced by bacterial and host ROS, has been observed in gastric epithelial cells upon infection as well. Abasic sites that are targeted by OGG1, such as 8oxodG lesions, are induced at a greater frequency upon *H. pylori* infection. Reduced expression of OGG1 allows for accumulation of abasic sites that would not be repaired by the other DNA repair pathways and lead to carcinogenic mutation-build up and cellular transformation (Kidane et al., 2014).

### Bacteria associated colon cancer

The association between bacterial infection and colon cancer has not been elucidated to the extent that *H. pylori* induced gastric cancer has been. Certain pathogenic species such as enterotoxigenic *Bacteroides fragilis* and *Escherichia coli* strain NC101 have been linked to colitis-associated colon cancer (Wu et al., 2009; Arthur et al., 2012), but no bacterial species has been proven to be a major causative agent in colorectal carcinogenesis. Rather, a collection of gram-negative and anaerobic bacteria has been observed in colorectal tumor tissues and may serve as a marker of cancer. The colon is home to commensal bacterial species that play various supporting and valuable roles in processes such as metabolism in the host. These microbiota have also been implicated in carcinogenesis and tumor formation in the colon, potentially through bacterial dysbiosis in the gut (McCoy et al., 2013; Warren et al., 2013).

One area of interest in this field is the presence of *Fusobacterium nucleatum* in abundance in colorectal tumor tissues (Kostic et al., 2013). *F. nucleatum* is a gram-negative microbe more often associated with the oral cavity. However, the bacterial species have been identified in the early stages of cancer in colorectal adenomas as well as carcinoma samples (McCoy et al., 2013). Introduction of *F. nucleatum* to mice was shown to speed up colonic tumorigenesis and induce a pro-inflammatory state through NF-κB signaling (Kostic et al., 2013). *F. nucleatum* has also been positively correlated with mortality linked to colorectal cancer, meaning that greater abundance of the bacteria more likely resulted in mortality due to the cancer (Mima et al., 2016). There is still much to study concerning whether *F. nucleatum* is a causative agent in colorectal carcinogenesis, but the preliminary studies have indicated the involvement of bacteria in the induction of cancer (Ray, 2011; Kostic et al., 2013; Gagnaire et al., 2017).

In the oral cavity, *F. nucleatum* is a very invasive bacterial species due to its ability to adhere well to mucous surfaces (McCoy et al., 2013). In the intestines, *F. nucleatum* can therefore act similar to *H. pylori* and adhere to host cell surfaces to alter cellular pathways with its bacterial proteins. Once *F. nucleatum* adheres to host cells, bacterial FadA adhesin binds E-cadherin to activate β-catenin signaling to increase cell growth and proliferation as well as to regulate inflammatory response of the cell (Rubinstein et al., 2013). *F. nucleatum* has also been found to activate TLR4 and ultimately lead to NF-κB activation (Yang et al., 2017). Many studies have linked *F. nucleatum* to a pro-inflammatory state (Kostic et al., 2013; Rubinstein et al., 2013). Inflammatory cytokine gene expression such as IL-10 and TNF- α has a positive association to abundance of *F. nucleatum* in the colon (Rubinstein et al., 2013). This also poses the possibility of *F. nucleatum* contributing to carcinogenesis and tumorigenesis by inducing genetic mutations as a result of prolonged inflammation and potentially the down-regulation of various DNA repair pathways, similar to what occurs in *H. pylori*-mediated inflammation leading to gastric cancer.

Other bacteria associated cancers: Other that *H. pylori* and *Fusobacterium, Salmonella typhi* infection has been associated with the development of gallbladder cancer (Mager, 2006; Di Domenico et al., 2017). All other bacteria associated with cancer is mentioned in the Table 1.

## Bacterial infection in cancer prevention and therapy

While a number of bacterial infections have been shown to increase the potential for carcinogenesis, recent developments in the field have provided evidence for a positive role of certain bacteria and toxins in cancer prevention and therapy (Mager, 2006). For example in one case-control study, researchers found that *Helicobacter pylori* infection correlated to a lower risk for esophageal cancer development (de Martel et al., 2005). Alternatively, the introduction of bacteria or its toxins to treat cancer has become a method of interest for many types of cancer. Dr. William Coley started to treat end stage cancers with a vaccine made of killed *Streptococcus pyogenes* and *Serratia marcescens* in the late 1800's to induce an initial fever followed by treatment for many different types of cancers (de Martel et al., 2005). More recently, researchers have shown that a vaccine with live attenuated *Salmonella enterica* serovar Typhi reduced tumor growth and enhanced survival in mice (Vendrell et al., 2011). The Bacillus Calmette-Guérin vaccine, which has a strain of *Mycobacterium bovis*, is used clinically for the treatment of high-risk urinary bladder cancer (Kucerova and Cervinkova, 2016). Finally, a number of bacteria species has been tried as anti-tumor agents in experimental models of cancer (Ryan et al., 2006).

## Future research directions

About 20% of the global cancer burden is linked to infectious agents including, but not limited to, *H. pylori, Hepatitis B* and *C* virus, and *Human papilloma virus* (Mantovani et al., 2008; Gagnaire et al., 2017). By studying how these infectious agents can lead to and exacerbate cancer states, we may be able to prevent certain cancers from forming or advancing and identify cancer markers or therapeutic targets in the treatment of cancer. It is already known that a chronic inflammatory state is able to induce DNA damage through oxidative stress induced by ROS production. As it is a hallmark of cancer, DNA damage must be repaired properly through the multiple machineries present within the cell (Colotta et al., 2009; Hanahan and Weinberg, 2011). However, along with inducing a chronic inflammatory state, bacterial infection may affect function and/or the level of DNA repair proteins leading to a buildup of genetic mutations in potential oncogenic and tumor suppressor genes that are major contributors to carcinogenesis. Studying the effect of infectious agents that are already linked to cancers, such as *F. nucleatum* and colorectal cancer, on the functionality of the various DNA repair pathways, can lead to novel identification of various markers for early cancer detection as well as more effective therapies and treatments that can combat the loss in DNA repair function in host cells.

## Author contributions

All authors listed, have made substantial, direct and intellectual contribution to the work, and approved it for publication.

### Conflict of interest statement

The authors declare that the research was conducted in the absence of any commercial or financial relationships that could be construed as a potential conflict of interest.
